# Supplementary material for: Mutations in PCYT2 disrupt etherlipid biosynthesis and cause a complex hereditary spastic paraplegia
Source: Brain. 2019 Oct 22;142(11):3382–97. doi: 10.1093/brain/awz291 (PMC6821184; doi:10.1093/brain/awz291)
Supplement: awz291_Supplementary_Data [file awz291_supplementary_data.zip › awz291-Suppl_data/Supplementary_Data4.zip!/Supplementary information 3 - fibroblast lipidomics.zip!/Fibroblast lipidomics/Controle vs Patient2/Statistical plots and data/PS[O] species/Random forest.pdf]

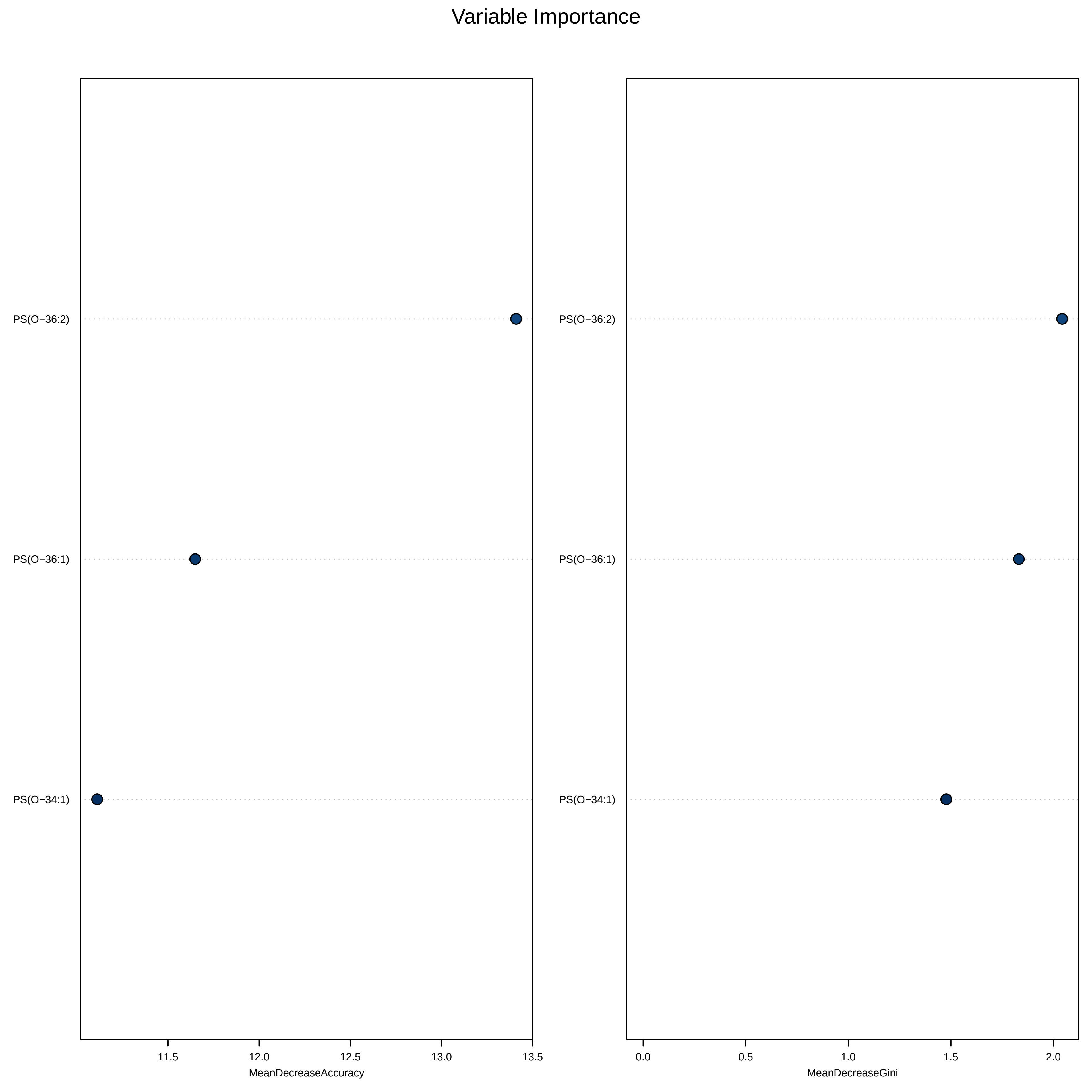

Variable Importance
PS(O−36:2)
PS(O−36:1)
PS(O−34:1)
11.5
12.0
12.5
13.0
13.5
MeanDecreaseAccuracy
0.0
0.5
1.0
1.5
2.0
MeanDecreaseGini
